# Bioproduction of the Recombinant Sweet Protein Thaumatin: Current State of the Art and Perspectives

**DOI:** 10.3389/fmicb.2019.00695

**Published:** 2019-04-08

**Authors:** Jewel Ann Joseph, Simen Akkermans, Philippe Nimmegeers, Jan F. M. Van Impe

**Affiliations:** ^1^BioTeC+, Chemical and Biochemical Process Technology and Control, Department of Chemical Engineering, KU Leuven, Leuven, Belgium; ^2^Optimization in Engineering Center-of-Excellence, KU Leuven, Leuven, Belgium; ^3^CPMF^2^, Flemish Cluster Predictive Microbiology in Foods, Leuven, Belgium

**Keywords:** thaumatin, natural sweetener, *Pichia pastoris*, sweet protein, recombinant proteins, systems biology

## Abstract

There is currently a worldwide trend to reduce sugar consumption. This trend is mostly met by the use of artificial non-nutritive sweeteners. However, these sweeteners have also been proven to have adverse health effects such as dizziness, headaches, gastrointestinal issues, and mood changes for aspartame. One of the solutions lies in the commercialization of sweet proteins, which are not associated with adverse health effects. Of these proteins, thaumatin is one of the most studied and most promising alternatives for sugars and artificial sweeteners. Since the natural production of these proteins is often too expensive, biochemical production methods are currently under investigation. With these methods, recombinant DNA technology is used for the production of sweet proteins in a host organism. The most promising host known today is the methylotrophic yeast, *Pichia pastoris*. This yeast has a tightly regulated methanol-induced promotor, allowing a good control over the recombinant protein production. Great efforts have been undertaken for improving the yields and purities of thaumatin productions, but a further optimization is still desired. This review focuses on (i) the motivation for using and producing sweet proteins, (ii) the properties and history of thaumatin, (iii) the production of recombinant sweet proteins, and (iv) future possibilities for process optimization based on a systems biology approach.

## Introduction

Food manufacturers are in constant search for alternative sweeteners for their products in accordance with the change of consumer perception on the intake of sucrose. Most of the times, the development of these alternatives poses certain difficulties in terms of meeting all the requirements for replacing sucrose. Due to the complex combination of properties existing within sucrose in terms of its taste and texture characteristics, it is often seen as a challenging task replace it with a low-calorie or non-caloric sweetener.

### Sugar

Sugars are extensively used ingredients in numerous applications especially in food and pharmaceuticals. It is a disaccharide which consists of one unit of glucose and one of fructose linked by glycosidic bonds and gets easily hydrolyzed into these simple sugars upon digestion. Due to the increased demand for sucrose, the production of these ingredients has elevated over the years and lingers as an important part of the diet worldwide.

The fragmentation of sugar into constituent monosaccharides occurs within the small intestine in the presence of the digestive enzymes and eventually gets transported into the bloodstream from the intestines. This leads to the elevation of blood glucose level until it gets transported by insulin (Pfeiffer et al., 2010). The breaking down of sugar is associated with its complexity, i.e., the more complex the sugar is, the higher the energy and longer the time required for it to break the linkages. Therefore, the elevation of the blood glucose level is dependent on the type of sugar consumed. Due to this effect on the blood glucose level, sugars can contribute to various adverse health effects. Bray and Popkin (2014) elaborate on such an outcome in their study by utilizing the results from clinical trials and meta-analyses. From their analyses and trials, it was observed that the consumption of sugar-sweetened beverages (SSBs) induced the risk of diabetes, metabolic syndrome and cardiovascular diseases and a reduction of weight gain could be achieved by minimizing the intake of these drinks.

Furthermore, several other studies also point toward the fact that high intake of sugars can instigate risk factors such as high blood pressure (He and MacGregor, 2015) and cardiovascular diseases (Johnson et al., 2009). Due to the high amount of energy that sugars provide to the body, they contribute to obesity as well. According to the findings of NCD-RisC (NCD Risk Factor Collaboration, 2016), it is estimated that obesity will be noticeably prevalent among adults worldwide with a possibility to increase further by 2025.

As a food ingredient, table sugar has played a paramount role until its value began to subside due to the public awareness which surfaced over the past years. This in turn triggered the development of alternative sweeteners that could effectively produce a similar effect in applications but have less impact on the overall health. The decrease in consumption of table sugar is evident from the data acquired on the changing trends in the consumption of low-calorie sweeteners (Sylvetsky and Rother, 2016). Moreover, the World Health Organization (WHO) now emphasizes the necessity for reducing added sugars in the diet and hence, recommends an intake of sugar less than 5% of the total caloric intake^1^.

### Sugar *Replacement*

The awareness on the health issues among the acknowledged has imposed the need for alternative solutions that have less impact on human health. Since the 1800s many sweeteners, both natural and artificial have been identified for various applications. The potency of these sweeteners is most often higher than that of sucrose.

**FIGURE 1:**
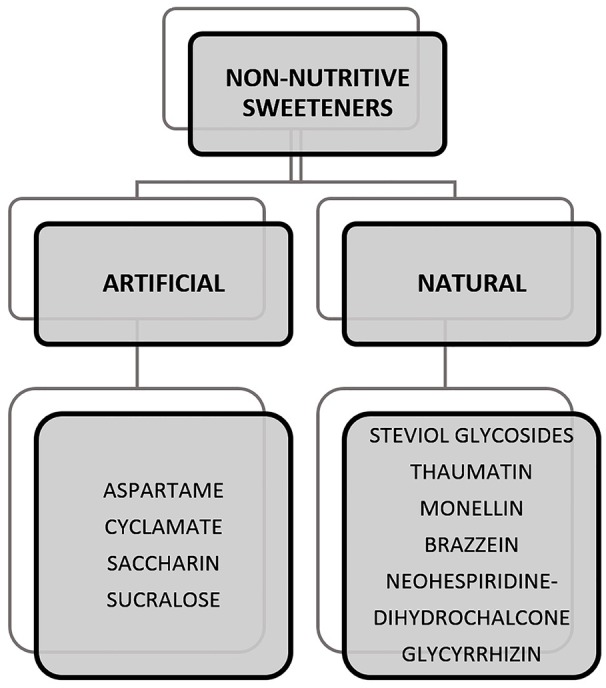
Examples of non-nutritive sweeteners (NNSs) that are artificially and naturally obtained (Carocho et al., 2017).

Sweeteners can be classified as caloric, low-calorie, and non-caloric. The caloric sweeteners are also known as nutritive sweeteners which provide energy in the form of carbohydrates. These can be seen naturally occurring in foods such as fructose in fruits or added to foods such as sucrose to drinks. This class of sugars also includes sugar alcohols such as sorbitol, lactitol, xylitol, mannitol, erythritol, trehalose, and maltitol. They provide calories which possibly contribute to health conditions on consumption in high quantities. In the case of sugar alcohols, consumption can also lead to other effects, i.e., irritable Bowel syndrome (IBS) and abnormal flatulence (Tuck et al., 2014; Yao et al., 2014). Specifically, erythritol, has the tendency to cause laxative effects when consumed in high quantities (Rzechonek et al., 2018). In this context, the rise of non-nutritive sweeteners (NNS) as an alternative ingredient surged within the market. Figure 1 provides a list of NNS that are artificially and naturally obtained and have been studied widely. Some of the NNS that are approved for use in food includes sucralose, aspartame, saccharin, acesulfame K, neotame, advantame, steviol glycosides, and luo han guo (monk fruit) extract out of which, the latter two are naturally derived NNS^2^. A more detailed discussion on artificial and natural NNS is provided in Section “Artificial Sweeteners” and “Natural Sweeteners.”

The selected alternative sweetener should withhold certain important properties that make it ideal for similar applications as sucrose. For instance, Bakal (1986) explains the prominent characteristics that an alternative sweetener is expected to hold when used as a substitute for sucrose. The author explains that in addition to being as sweet as sucrose, it must also possess similar functional and physiological properties. Sweetness factor is an inevitable criterion when finding substitutes for sucrose especially because the potency value of these sweeteners is defined with respect to it. Besides this, the substitutes are also expected to provide a sensory profile which is similar to that of sucrose. However, some authors claim it to be very challenging for acquiring similar taste characteristics to the distinctive taste profile of sucrose (Larson-Powers and Pangborn, 1978; Palazzo and Bolini, 2013). Sucrose also plays a paramount role in delivering specific properties such as structure, texture, and flavor (Koeferli et al., 1996; Pareyt et al., 2009) in various applications making it further distinctive in nature. Moreover, Hough (1997) emphasizes properties such as solubility, chemical and thermal stability, compatibility with production and applications, ease of production, non-toxicity and being inexpensive.

### Artificial Sweeteners

Artificial sweeteners can be described as non-caloric or low-calorie sweeteners because they provide very few calories when compared with sucrose due to lesser absorption by the digestive system. Unlike the caloric or nutritive sweeteners, they provide no calories or hardly any calories but possess a much higher sweetness than sucrose. These intense sweeteners are usually mixed with bulking agents such as polydextrose and maltodextrin to enhance their applicability. Although the influence of artificial sweeteners on health aspects has been discussed widely in the past, there are still some uncertainties revolving around these sweeteners in terms of usage limitations (Marti et al., 2008). Examples of this class are acesulfame-K, aspartame, neotame, saccharin and sucralose, which are approved by the United States Food and Drug Administration (FDA) and the European Food Safety Authority (EFSA). The sweetness index of these sweeteners is included along with their E numbers in Table 1.

**Table 1:** The major artificial sweeteners used in the food and pharmaceutical industry (Lindley, 2012; Chattopadhyay et al., 2014).

Sweetener	Sweetness index	E Number
Acesulfame	200 x	E 950
Aspartame	180–200 x	E 951
Cyclamate	30–50 x	E 952
Saccharin	300 x	E 954
Sucralose	600 x	E 955
Alitame	2000 x	E 956
Neotame	7,000–13,000 x	E 961
Advantame	20,000 x	E 969

Out of these artificial sweeteners, aspartame is a prevalent ingredient around the globe and is used in a considerable number of food products (Myers, 2007). Despite the benefit of using it as an alternative to sucrose in numerous applications, the safety aspects still remain controversial. For instance, some health effects such as dizziness, headaches, gastrointestinal issues, and mood changes are associated with aspartame. These health effects are elaborated in the study of Whitehouse et al. (2008). Ferland et al. (2007) investigated the effect of this sweetener on the glucose and insulin levels in blood plasma. The target group selected for the study were men diagnosed with type 2 diabetes and the influence of aspartame was assessed during acute exercise. During their investigation, it was noticed that the consumption of aspartame led to an increase in glucose and insulin levels similar to that of sucrose. Pearlman et al. (2017) emphasizes that the artificial sweeteners have an impact on host microbiome, gut-brain axis, glucose homeostasis, energy consumption, overall weight gain, and body adiposity that is evident through the profound amount of available data. With regard to the microbiota, a study conducted by Suez et al. (2014) claims that the usage of non-caloric artificial sweeteners can boost the risk of glucose intolerance.

Apart from this, several other studies on artificial sweeteners have developed severe concerns among the consumers regarding its usage. For instance, these artificial sweeteners can enter the environment and undergo degradation thereby resulting in toxic products. The authors Kokotou et al. (2012) summarize the impact of these ingredients as environmental pollutants. In their review article, they elaborate on the findings related to the environmental impact of artificial sweeteners along with the available methodologies that can be incorporated for detection of the trace compounds. While NNS are widespread today for numerous applications, with respect to the approval from FDA and other studies, the safety aspects regarding their regular usage remain uncertain^3^. It is therefore not surprising that the World Health Organization (2015) predicts an increase in the production of natural alternatives based on the shift in consumer preferences toward more natural products.

### Natural *Sweeteners*

In this context, the plant-derived sweeteners such as stevioside, glycyrrhizin, osladin, etc. come into the limelight with the added advantage of not imposing any health issues. For example, stevia which is a natural sugar substitute was not seen to increase the blood glucose levels compared to sucrose with the findings of Anton et al. (2010). During this study, the effect due to the intake of stevia, aspartame, and sucrose were compared. The study concluded that stevia reduced blood insulin levels significantly compared to both sucrose and aspartame. This demonstrates the health benefit of a natural sweetener compared to an artificial sweetener.

Apart from the previously mentioned sweeteners, there are several sweet compounds that are found in nature belonging to three classes namely terpenoids, flavonoids, and proteins (Kinghorn and Compadre, 2001; Fry, 2012). Most proteins do not necessarily elicit any sweet taste and flavor. However, this is not the case with the sweet proteins derived naturally from plants that are grown in tropical regions of mostly Africa and Asia. These naturally derived proteins are very sweet, i.e., even 100–1000 times sweeter than table sugar or sucrose on a mass basis. Such sweeteners which are low-calorie or non-caloric in nature can be used to substitute sucrose in sugar-based food and drinks thereby posing a solution for those who are prone to diabetics or obesity. They come with the advantage of not imposing significant health concerns compared to the previously mentioned artificial sweeteners.

Until now, eight sweet proteins are identified namely, thaumatin (Van der Wel and Loeve, 1972), monellin (Inglett and May, 1969), mabinlin (Liu et al., 1993), lysosyme (Maehashi and Udaka, 1998), pentadin (Faus, 2000), brazzein (Ming and Hellekant, 1994), curculin (Harada et al., 1994), and miraculin (Takahashi et al., 1990). These proteins are isolated mostly from tropical plants with an exception for the protein lysozyme, which is derived from egg whites. The research in sweet-proteins has been ongoing for several years now and some of the major characteristics of each protein have been studied over the years. The most important characteristics of these proteins are summarized in Table 2. Of these eight proteins, thaumatin is the most developed, commercialized and regulated sweet protein (García-Almeida et al., 2018).

**Table 2:** Comparison on the characteristics of different sweet proteins.

	Sweetness
Sweet protein	(weight basis)	Amino-acids	Properties	Natural Source	Bioproduction host
Thaumatin	3000 x	207	• Taste slightly differs from sucrose • used as flavor modifier • Stable at 120°C; withstand pasteurization and UHT process • EU approved (E957)	*Thaumatococcus daniellii* Benth	Bacteria^1,2,3^ yeast^4,5,6^ Fungi^7,8^ Transgenic plant^9,10^
Monellin	3000 x	45 (A chain)/50 (B chain)	• Intense sweetness • Loss of sweetness above 50°C	*Dioscoreophyllum cumminsii* Diels	*E. coli*^11^ Yeast^12^ Transgenic plant^13^
Brazzein	2000 x	54	• Heat and pH stable; 100°C for 2 h • Tastes similar to sucrose • Shorter aftertaste • Water soluble • Works well with stevia	*Pentadiplandra brazzeana* Baillon	*S. cerevisiae*^14^ *E. coli*^15^
Neoculin/curculin	550 x	114	• Degrade at 50°C	*Curculigo latifolia*	*E. coli*^16^ *Aspergillus oryzae*^17^
Mabinlin	100 x	33 (A chain) 72 (B chain)	• 4 Variants • Mab II unchanged at 80°C for 2 hrs • 1, 3, and 4 not stable	*Capparis masaikai* Levi	*E. coli*^18^ Cultured plant cells^19^
Pentadin	500 x	54	• Rapid loss of sweetness	*Pentadiplandra brazzeana* Baillon	–
Miraculin	N. A	191	• Modifies sour taste into sweet taste	*Richadella dulcifica*	*E. coli*^20^ Transgenic plants^21^

*^*1*^(Faus et al., 1996); ^*2*^(Illingworth et al., 1988); ^*3*^(Illingworth et al., 1989); ^*4*^(Edens et al., 1984); ^*5*^(Edens and van der Wel, 1985); ^*6*^(Cregg et al., 2000); ^*7*^(Hahm and Batt, 1990); ^*8*^(Faus et al., 1998); ^*9*^(Witty, 1990); ^*10*^(Bartoszewski et al., 2003); ^*11*^(Chen et al., 2005); ^*12*^(Kim and Lim, 1996); ^*13*^(Peñarrubia et al., 1992); ^*14*^(Guan et al., 1995); ^*15*^(Assadi-Porter et al., 2000); ^*16*^(Suzuki et al., 2004); ^*17*^(Nakajima et al., 2006); ^*18*^(Gu et al., 2015); ^*19*^(Xiong and Sun, 1996); ^*20*^(Kurihara, 1994); ^*21*^(Sun et al., 2006).*

One of the most crucial factors to take into account while considering sugar replacers is the sweetness. The sweetness factor is measured with reference to sucrose. Sweetness perception occurs when sugar dissolves with the saliva and bind with the receptors on the tongue. Therefore, humans perceive sweet taste through the T1R2–T1R3 receptor which are heterodimers belonging to the family of G-protein-coupled receptors (GPCRs) (Margolskee, 2001; Nelson et al., 2001; Li et al., 2002). These receptors which have several binding sites (Vigues et al., 2008) get activated when the compounds that elicit sweet taste bind to it. However, the binding property for each sweetener is different and hence, all the sweeteners do not necessarily bind at the same sites (Fernstrom et al., 2012). This leads to the varying perception of sweetness of the different proteins. Table 3 lists some of the most important properties of bulk Thaumatin.

**Table 3:** Properties of bulk thaumatin.

Parameter	Specifications
Visual appearance	Yellow to light tan powder
Solubility (water)	600 mg/mL
pH	2.7–6.0
Stability (dry concentrate at <10°C)	>6 months
Minimum total thaumatin content	≥98% HPLC

*www.fda.gov/downloads/Food/IngredientsPackagingLabeling/GRAS/NoticeInventory/ucm592042.pdf.*

### Review *Outline*

The increasing awareness among the public over the adverse effects of sucrose and artificial sweeteners is creating opportunities for the exploration of naturally derived alternatives, such as plant-derived sweeteners. In this review, the advances in the production of the sweet-protein thaumatin will be discussed. Section “Thaumatin” summarizes the current relevance of thaumatin based on its characteristics and possible applications. Also, a detailing will be done on the aspects related to the production of this protein via the various routes that have been studied. Section “Bioproduction” focuses on the bioproduction aspects involved in the production of thaumatin with a focus given to the selection of strains, DNA construction for protein expression, production process and downstream processes. The final section summarizes on the possibility to integrate knowledge from systems biology for the optimization of thaumatin bioproduction.

## Thaumatin

Thaumatin, a sweetener desired in the market today, is naturally derived from the fruit arils of a tropically grown plant called *Thaumatococcus daniellii* (Benth) belonging to the family Marantaceae (Kant, 2005). *Thaumatococcus daniellii* is a large flowering herb which can grow up to 4 m high and is commonly found in the rainforests of West Africa ranging from Sierra Leone to the Democratic Republic of Congo. The fruit was called ‘katemfe’ or ‘miraculous fruit of Sudan’ (Daniell, 1855; Inglett and May, 1968; Van der Wel, 1974). They are also known as miracle fruit, miracle berry (Wiersema and León, 1999), Yoruba soft cane and African serendipity berry.

### Characterization of *Thaumatin*

The sweetness potency of thaumatin was first elucidated by a British surgeon, Daniell (1855). It elicits a sweetness 100,000 times higher than that of sucrose on a molar basis even at a low concentration of 50 nM. It is a caloric sweetener; however, it has a negligible impact at the level of concentration used within applications. It consists of a single-chain of 207 amino acid residues and has a relative molecular mass of 22 kDa (Van der Wel and Loeve, 1972). The stability of the protein is dependent on the matrix. For instance, the sweetness of the protein is retained when boiling at a pH below 5.5 for 1 h. It has even been observed that at these pH values the sweetener is stable during heat processes such as pasteurization, canning, baking and ulta-high-temperature processing (Gibbs et al., 1996; Lord, 2007). However, when heated above 70°C at a pH of 7.0, aggregation and loss of sweetness were observed (Mcpherson and Weickmann, 1990; Kaneko and Kitabatake, 1999). The loss of sweetness can be associated with heat denaturation or breakage of disulfide bridges (Higginbotham, 1979) within the protein. The heat and acid stability of the thaumatin molecule is due to the tertiary structure which is stabilized by the eight disulfide bridges (Van der Wel and Ledeboer, 1989).

**FIGURE 2:**
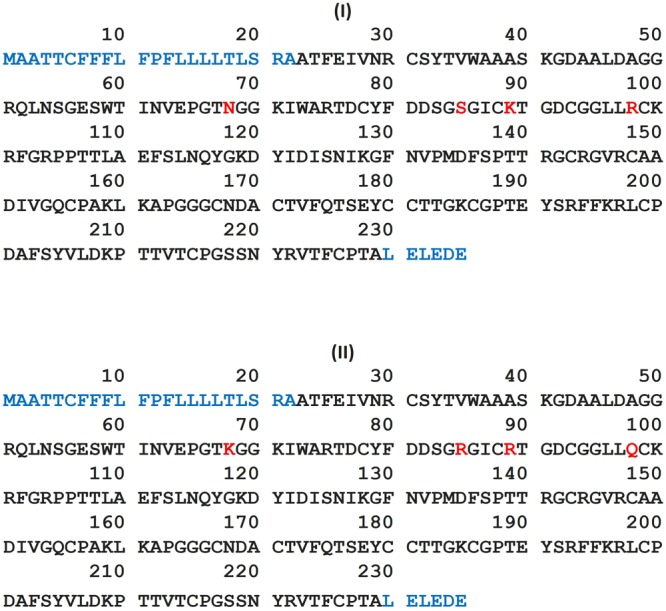
Amino acid sequence of the two main forms, thaumatin I and thaumatin II www.uniprot.org. Blue characters denote the signal peptide and red characters denote the differences in the sequences at four positions.

It is important to know that different forms of thaumatin have been identified. The amino acid sequences of the two main forms, thaumatins I and II were originally reported by Iyengar et al. (1979) and Edens et al. (1982). A correction was made to the reported sequence for thaumatin I by Kaneko (2001). The two sequences are presented in Figure 2. Both forms have been expressed in microbial hosts. When studying the thaumatin sequences, Lee et al. (1988) assigned the two prominent variants as thaumatin A and thaumatin B. Thaumatin A later on appeared to be the same form as thaumatin I.

Numerous novel sweeteners have been introduced in the market that are expected to play as a substitute for sucrose by possessing similar sensorial properties as well as being safe. Prior to the introduction of these substitutes in the market, the laws and directives regulated by the authorities such as the FDA and EFSA must be followed. However, the rules and regulations differ throughout the world, which makes the immediate introduction of these ingredients into the market more difficult. For those that are approved to be used, the regulatory bodies such as FDA, Scientific Committee for Food of the European Commission (SCF), or Joint Expert Committee on Food Additives (JECFA) of the FAO/WHO have provided an acceptable daily intake. Considering the health aspects related to thaumatin, it does not instigate any tooth decay and can be suitable for the diabetic, unlike artificial sweeteners (Kinghorn et al., 1998). Moreover, the metabolism of this sweetener is analogous to other dietary proteins. The study conducted by Hsu et al. (1977) shows that digestion of thaumatin occurs more rapidly than egg albumin. Additionally, several studies involving the safety aspects of thaumatin indicate that the sweetener does not cause any allergenicity or toxicity. Numerous studies have been conducted to assess the toxicity of thaumatin. For instance, the Joint FAO/WHO Expert Committee on Food Additives, Food and Agriculture Organization of the United Nations & World Health Organization (1986) report claims that the protein is free from any toxic, genotoxic, or teratogenic effect. There is substantial evidence from various research, demonstrating that thaumatin is not an allergen to oral mucosa (MacLeod et al., unpublished) and on other treatment associated allergic effects (Eaton et al., 1981). The authors Higginbotham et al. (1983) also denote that thaumatin does not cause any hazardous effect when used as a flavor modifier or partial sweetener within a specific level of consumption. The safety of this protein was evaluated by SCF and JECFA who concluded that thaumatin can be considered as an acceptable ingredient for use (European Food Safety Authority (EFSA) Panel on Food Additives and Nutrient Sources Added to Food, 2015). This sweet protein is approved within the European Union since 1984 (E957) under Annex II of Regulation (EC) No. 1333/2008 (European Food Safety Authority (EFSA) Panel on Food Additives and Nutrient Sources Added to Food, 2015) and possesses GRAS (Generally Regarded as Safe) approval in the United States (FEMA GRAS Number 3732). It was approved in Great Britain in 1983 to be used in food and pharmaceuticals with an exception for baby foods. It is also allowed as a flavor enhancer and high-intensity sweetener within several countries (Zemanek and Wasserman, 1995). The FEEDAP (2011) also signals the safety of the protein for animals and approves its use as an additive within a level of 1 to 5 mg/kg. Some of the characteristics of thaumatin are listed in Table 4.

**Table 4:** Recombinant thaumatin produced in different organisms.

Organism	Promoter	Secretion	Yield	Study
*E. coli*	Tryptophan/lactose	No	Low	Edens et al., 1982
*S. cerevisiae*	3-Phosphoglycerate	No	Low	Lee et al., 1988
*B. subtilis*	A-Amylase	Yes	1 mg/L	Illingworth et al., 1988
*S. lividans*	B-Galactosidase	Yes	0.2 mg/L	Illingworth et al., 1989
*A. awamori*	Glucoamulase	Yes	5–7 mg/L	Faus et al., 1998

Apart from being a low-calorie sweetener, thaumatin can also act as a flavor modifier in food applications. Hence, such unique properties of this sweet-protein make it an attractive ingredient for the food industry. The major applications of thaumatin are seen as an additive in chewing gum, dairy, pet foods, and animal feeds (Smith and Hong-Shum, 2011). It also qualifies as a sweetener in other food products such as ice-creams and sweets within a permitted level of 50 mg/kg. In soft drinks and dairy products, it is mostly utilized as a flavor enhancer within a limit of 0.5 mg/L and 5 mg/kg respectively (Mortensen, 2006). The application of thaumatin is not just limited to imparting sweetness in products but also enhancing flavor and masking undesirable notes in food and pharmaceuticals (Etheridge, 1994). The onset of sweetness for this protein is slower and often results in a licorice aftertaste. Due to this residual effect, thaumatin is often applied in combination with other sugar substitutes.

### History of *Thaumatin Production*

**FIGURE 3:**
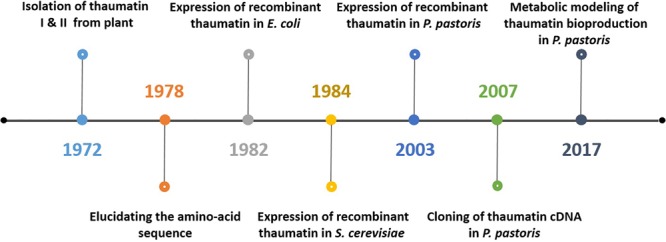
Schematic representation of the most significant events in thaumatin production starting from naturally occurring plants toward microbial production and the current integration of systems biology.

Bioprocessing plays a significant role in biotechnology. The advancement of molecular biology and recombinant DNA technology facilitates the production of human proteins through heterologous expression within microorganisms. For instance, human insulin, which is important to treat diabetes mellitus, was initially purified from bovine and porcine pancreas extracts. The economic factor associated with its production combined with the immune responses caused in patients due to the animal insulin has instigated the implementation of heterologous expression using *Escherichia coli* (Jozala et al., 2016). The series of activities involved in the production of thaumatin follows a similar trend. Figure 3 schematically represents how the production of thaumatin evolved from the traditional plant extraction to a more relevant microbial production today.

Initially, thaumatin was produced in 1972 by van der Wel and Loeve from the fruits of *Thaumatococcus danielli* through aqueous extraction. During this study, the authors examined the protein content from the fruit at various maturation stages and compared the amount of different forms of thaumatin such as thaumatins I and II. Both the forms were found to be similar with respect to their amino acid sequences. It was elucidated from the study that they have the same N-terminal amino acid alanine and molecular weights of 21000 ± 600 and 20400 ± 600 Da. Their findings also included the relation between the protein content in the fruit and the region of cultivation. During the study, a total of 4900 mg of extract per 2700 g of fruit was obtained (0.18%). The sweetness of the aqueous solution of thaumatin obtained during the experiment was seen to decrease when heated above 75°C and also at a pH below 2.5 at room temperature. Korver et al. (1973) further investigated these two main forms of thaumatin. The study was to foresee the conformational changes undergone by the protein due to heat denaturation. It concluded that the loss of sweetness of the protein during the application of heat was basically due to the irreversible heat-induced conformational transition. The primary structure of thaumatin was unraveled by Iyengar et al. (1979) to gain a better understanding of the sweetness mechanism of the protein.

The product attained upon aqueous extraction from the Katemfe fruit was first found as a mixture of proteins. In the following year thaumatin I was crystallized from which the physical attributes and diffraction data of the crystals were attained (van der Wel et al., 1975). Although the protein can be recovered from the extract through selective ultra-filtration, the final product available commercially still consist of some impurities. Hence, the commercially available thaumatin sweetener is a mixture of thaumatins I and II along with other traces of the source material such as arabinogalactan and arabino glucuronoxylan polysaccharides (European Food Safety Authority (EFSA) Panel on Food Additives and Nutrient Sources Added to Food, 2015).

## Bioproduction

Due to the increasing awareness of the public over artificial sweeteners, natural sweeteners gained popularity. For instance, thaumatin is favored by the public to replace sucrose in food products. However, the fact that thaumatin is produced from a tropically grown plant limits its availability while the demand is high. Moreover, the production process can be greatly affected with respect to the source material availability and quality. In order to attain a more stable production of the protein to meet the demand, a series of studies has been performed involving its production through genetically engineered microorganisms and transgenic plants (Nabors, 2001; Jain and Grover, 2015; Masuda, 2016).

Commercially, proteins can be produced using techniques such as genetic and protein engineering. These proteins are used by the biopharmaceutical industry, enzyme industry and agricultural industry within the fields of medicine, diagnostics, food, nutrition, etc. In the early 1900s, the microbial fermentation industry came into the scene through the production of chemicals such as acetone, butanol, and citric acid. The first protein pharmaceutical that was produced through recombinant DNA technology and approved by the FDA was Human Insulin, in 1982. Some examples of other recombinant proteins are albumin, human growth hormone (HGH) and factor VIII. Furthermore, advancement in the technologies for production processes has contributed to the growth of the recombinant protein market. For example, the evolution in mammalian cell expression, baculovirus expression, *E. coli* expression and bioreactor systems have facilitated the ease of production of these proteins. Currently, more than 400 protein drugs achieved from recombinant technologies have been approved and marketed worldwide and more than 1300 of them are undergoing the process of attaining approval (Global Data, 2015)^4^. In 2016, the recombinant protein market witnessed a value of US$ 347.2 million globally. According to information provided by the Coherent Market Insights company (2018), a compound annual growth rate of 6.2% is expected for the period 2017–2025^5^.

Numerous factors have to be considered for an efficient and low-cost production of recombinant proteins. Understanding the mechanism and metabolic pathway of thaumatin production can help in improving the yield and productivity. Since this protein has great potential as a substitute for the less safe sweeteners or sugar alternatives, an improved and commercially viable production is required. However, there is still much work to be done for the development of novel methods that can be utilized for the production of such recombinant proteins.

### Host *Microorganisms*

The two major factors that need to be taken into account for the expression of recombinant proteins are cloning of the desired DNA and the amplification of the protein in the chosen expression system. Selection of the expression system can be based on certain characteristics such as; quality of the protein, functionality, productivity, and yield (Demain and Vaishnav, 2009). Also, for the production of thaumatin, a suitable expression system needs to be chosen. Several systems are available for protein expression such as bacteria, yeasts, molds, mammals, plants or insects, transgenic plants, and animals. A wide number of studies has already been reported on the expression of thaumatin in microorganisms Several of these studies reported that the protein yield was low and that the attained product was inactive (Edens et al., 1982; Edens and van der Wel, 1985; Illingworth et al., 1988, 1989; Lee et al., 1988; Hahm and Batt, 1990; Faus et al., 1997). In other cases, a higher yield could be attained through the use of artificial genes and codon optimization. For example, in Daniell et al. (2000) an acceptable yield could be achieved through the utilization of artificial genes with optimized codon usage encoding thaumatin II. This required a renaturation process because the recombinant proteins were attained as inclusion bodies which were insoluble and inactive. As such, the use of recombinant microorganisms is definitely not a straightforward route and considerable research has been undertaken for the purpose of optimizing the recombinant production of thaumatin.

An approach toward the usage of transgenic plants has also been tested in a number of studies for the synthesis of sweet proteins. The usage of plant cells or tissue culturing has certain advantages in various applications such as (i) post-translational modification similar to mammalian cells, (ii) no health issues on humans and (iii) simplified large-scale production (Doran, 2000; Hellwig et al., 2004). However, it has certain drawbacks when compared to the microbial production. For instance, the attempts on producing sweet-proteins especially, thaumatin in potatoes, strawberries, etc. showed a lower level of accumulation. A sufficient amount of the recombinant thaumatin is achievable from transgenic barley and tomato. However, there is significant scope for improvement regarding the extraction and purification of the protein (Firsov et al., 2016). Even though the usage of plant system has shown certain advantages over microbial systems in terms of scalability, economy and safety, they still lack some benefits attainable from the microbial hosts such as the possibility to control the growth conditions and product consistencies throughout the batches. Additionally, a further improvement on protein stability and recovery is required (Hellwig et al., 2004). Moreover, considering some of the potential issues associated with the use of transgenic plants, such as allergic reactions and regulatory uncertainties, limits it from a wider application. As such, the following sections are limited to the production of recombinant thaumatin using microorganisms as a host.

#### Bacteria

*Escherichia coli* is one among the earliest and most commonly used host organism for protein expression (Terpe, 2006) due to the fast growth and expression, ease of culture and high yields (Swartz, 1996). Moreover, the genetics of *E. coli* are well-understood when compared to other organisms. Despite its great advantages, *E. coli* has certain drawbacks that can possibly influence the production efficiency of recombinant proteins. This is because a high cell density of this organism results in large quantities of acetate, which is toxic to cells. It is also reported through studies that this organism fails to produce very large proteins. Additionally, some issues were observed for *E. coli* system in terms of difficulties in producing proteins that have disulfide bonds and refolding ability. Another factor is the failure to produce modified protein due to the absence of glycosylation (Jenkins and Curling, 1994). Glycosylation affects properties such as solubility, stability functionality, immunogenicity, etc. In order to achieve glycosylation for attaining a stable and properly folded protein, a higher advantage is noticed for recombinant production in yeast, mold, insect or mammalian cells. A solution can be opting for secretion of the heterologous protein into the medium instead of intracellular inclusion bodies. This way, soluble and active proteins can be attained with a much easier downstream processing and cost reduction (Mergulhão et al., 2005). Emergence of other bacterial systems can also be noticed over the years in the field of recombinant protein production. The engineering of *Lactococcus lactis*, a gram-positive bacterium for membrane protein expression is one such example (Chen, 2012). Additionally, they also possess advantages over *E. coli* in terms of *being* GRAS and endotoxin free (Yeh et al., 2009). *Pseudomonas* species such as *P. fluorescens, P. aeruginosa*, and *P. putida*, were also found as suitable alternatives to *E. coli* expression systems to attain higher yields of the recombinant protein. Today, several other bacterial systems are widely explored as cell factories. The authors Ferrer-Miralles and Villaverde (2013) have summarized in their paper the most important bacterial hosts that can be utilized as cell factories for recombinant protein production.

The thaumatin II gene has been cloned into *E. coli* K12 by Edens et al. (1982). However, this resulted in a very low production of the protein. Later, Faus et al. (1996) managed to express a synthetic gene encoding the same DNA sequences of thaumatin II successfully in *E. coli*. An immunoblotting analysis confirmed that the expressed thaumatin had a similar molecular weight as that of the plant source. Followed by this, an attempt by Daniell et al. (2000) replicating the same system resulted in approximately 40 mg of purified thaumatin. This product showcased a similar threshold value of sweetness as that from the natural origin.

#### Fungi

A myriad of studies is available on bioproduction activities using *Aspergillus* and *Escherichia* species. However, the utilization of a yeast and other fungi species is found predominantly favorable due to the complexity in the growth of *Aspergillus* and lower yield in *Escherichia*. Considering the two most utilized organisms for the production of these relevant proteins; *Saccharomyces cerevisiae* and *Pichia pastoris*, both have the capability to produce proteins that are larger than 50 kDa in high yields and a possibility for glycosylation. Yeasts have the ability to produce chaperonins that can help in the folding of certain proteins and can handle S–S rich proteins (Demain and Vaishnav, 2009). With respect to thaumatin production, yields higher than 100 mg/L have been attained from yeast (Masuda and Kitabatake, 2006).

Gellissen et al. (2005a) emphasizes that yeast has more favorable conditions over other eukaryotic and prokaryotic systems to produce recombinant proteins thereby, resulting in yields of multigram range. The compact genome of yeasts makes the gene identification process much simpler (Goffeau et al., 1996). Moreover, they are robust, fast growing with a short lifecycle of 90 min and can be easily manipulated. Yeasts are easy to use for fermentations involving rapid growth into high cell densities on simple media. They also possess certain safety aspects by not involving with pathogens, viral inclusions, or pyrogens. Moreover, these eukaryotes have the ability to secrete and modify proteins (Gellissen et al., 2005b). It also comes with the advantage of DNA transformation facilitating gene cloning and genetic engineering. Thanks to all of these advantages, the application of yeast expanded widely from the chemical and enzyme production to the production of biopharmaceutical ingredients.

##### Saccharomyces cerevisiae

One such yeast, *S. cerevisiae*, has been utilized as an ideal organism for several biological studies. For instance, the ethanol industry prefers this yeast for fermentation of raw materials such as sugar cane and beets or corn and wheat due to its high industrial potential. *S. cerevisiae*, commonly known as Baker’s yeast, is one of the most industrially relevant eukaryotic microorganisms, mainly for bioproduction due to its availability, compatibility and more importantly, knowledge access of its genetic and physiological background. The genetic manipulation of this organism is comparatively easy and also widely accessible due to the available collections of genetic tools. Moreover, they exhibit a fast growth within a protein-free media, possess the capability for post-translational modifications and extracellular secretion (Tyo et al., 2014; Tang et al., 2015). Due to these properties of the organism, they have been widely used for bioprocesses and recombinant protein expression (Mattanovich et al., 2014). Such a platform is a prerequisite for the expression and production of heterologous proteins within the microbial system. However, while selecting an expression host it is important to note that overexpression of recombinant proteins can lead to intracellular accumulation and reduction of yields (Idiris et al., 2010; Tyo et al., 2014). It has to be taken into account that *S. cerevisiae* organism also possesses certain limitations. For example, Gellissen et al. (2005a) details that this microorganism has the tendency to hyperglycosylate recombinant proteins and results in batch inconsistencies due to strain instabilities caused by the use of episomal vectors.

The expression of preprothaumatin in *S. cerevisiae* was demonstrated by Edens et al. (1984) using a promoter fragment of the glyceraldehyde-3P-dehydrogenase (GAPDH) gene. Another yeast, *Kluyveromyces lactis* was also expressed with recombinant thaumatin II but a lower secretion of the protein was observed (Edens and van der Wel, 1985). An alternative solution to overcome these limitations can be by utilizing the non-conventional yeasts which have started gaining interest over the past decades (Reiser et al., 1990). This class of yeast includes mainly *P. pastoris* (Ilgen et al., 2005), *Hansenula polymorpha* (Gellissen, 2000, 2002; Kang and Gellissen, 2005), *Candida boidinii* (Sakai et al., 1996), *Pichia methanolica* (Raymond et al., 1998), *Arxula adeninivorans* (Böer et al., 2005), *K. lactis* (Gellissen and Hollenberg, 1997), and *Yarrowia lipolytica* (Madzack et al., 2005). These methylotrophic yeasts have the ability to utilize methanol as a sole carbon source for carbon and energy. The growth associated with the utilization of methanol is possible due to the presence of alcohol oxidase that is not included in the glucose-grown cells. Out of these, *H. polymorpha* and *P. pastoris* have been extensively used as academic tools and for production of proteins that are commercially available today.

##### Pichia pastoris

Given the specific successes that have been obtained with the production of recombinant thaumatin in *P. pastoris*, this microorganism is discussed here in more detail. Similar to *S. cerevisiae, P. pastoris* also has a pertinent fermentative growth. *P. pastoris* was first isolated from the chestnut tree in 1919 and was described as *Zygosaccharomyces pastori* (Guilliermond, 1920). However, in the 1950s different strains related to this yeast were isolated from Oak trees by Herman Phaff and he renamed it as *P. pastoris* (Phaff et al., 1956). Close to 10 years back, *P. pastoris* was re-classified into the genus *Komagataella* and since then given the name *Komagataella phaffii*. The protein production platforms utilized today are technically speaking either *K. phaffii* or *K. pastoris*, which are the same (Kurtzman, 2009).

A further classification can be considered for the *P. pastoris* strains. This can be wild-type strains (X-33, Y-11430), auxotrophic mutants that are defective in histidinol dehydrogenase (e.g., GS115), mutants that are defective in genes involved in methanol utilization (KM71, MC 100-3) and protease-deficient strains (SMD1163, SMD1165, SMD1168) (Jahic et al., 2006). *P. pastoris* is a yeast belonging to an integral part within the field of bioproduction for the past years and is foreseen to remain significant also in the future. It is a widely used single-celled organism for recombinant protein production due to the advantages it exhibits compared to other microorganisms. Moreover, it is a Crabtree-negative yeast meaning that it has preference for respiration rather than fermentation. Unlike Crabtree-positive yeasts, they do not use up the carbon source by producing ethanol and instead facilitate the production of higher biomass constituting toward more recombinant production. This makes *P. pastoris* more suitable for recombinant protein production than Crabtree-positive yeasts such as *S. cerevisiae*.

All strains of *P. pastoris* are derived from the wild-type NRRL Y-11430 (Northern Regional Research Laboratories) (Macauley-Patrick et al., 2005). In addition to sharing a similar intracellular environment and post-translational protein processing capacities with higher eukaryotes, the proteins generated from these strains are presumably correctly processed. Expression of recombinant proteins in the methylotrophic *P. pastoris* offers the possibility for an easier and faster production of high quantities of the protein. Usage of such a system facilitates the construction of heterologous protein in a more straightforward manner. This can be mainly attributed to the ease of genetic manipulation of this species and its fast growth on inexpensive media resulting in high cell densities. Moreover, this yeast has the capability to perform post-translational modifications including protein folding, proteolytic processing, disulfide bond formation and glycosylation (Cereghino et al., 2002). Additionally, it comes with the advantage of simple a purification process due to the low levels of native proteins that gets secreted (Cregg et al., 1993).

*Pichia pastoris* also has the capability to produce from milligram-to-gram quantities of proteins, making it suitable for both laboratory research and industrial manufacturing (Hellwig et al., 2001). Hence, this yeast has been utilized as an ideal host for higher production of different recombinant proteins (Tuite et al., 1999; Cregg et al., 2000; Cereghino and Cregg, 2000; Cereghino et al., 2002; Macauley-Patrick et al., 2005). From past literature, it can be observed that this strain has been successfully utilized as a host for more than 600 recombinant proteins (Cereghino and Cregg, 2000; Macauley-Patrick et al., 2005). From the discussion provided by the various authors it can be understood that *P. pastoris* plays a significant role in the production of recombinant proteins (Cregg et al., 2000; Cereghino and Cregg, 2000; Abad et al., 2010), especially for the production of complex proteins which need post-translational modifications and those with disulfide bridges (Cereghino et al., 2002). Therefore, more attention will be given to the bioproduction of thaumatin with *P. pastoris* in the remainder of this review.

##### Filamentous fungi

Filamentous fungi have placed a mark in industrial bioproduction due to their ability to produce primary metabolites such as organic acids, human therapeutics, fungal enzymes, and single cell protein. As such, the production of recombinant proteins is not just limited to bacteria and yeasts. Filamentous fungi have the capability to grow at high rates and densities in the presence of inexpensive media using simple fermenters. *Aspergillus awamori* was also utilized for attaining a higher yield of recombinant thaumatin which resulted in a non-homogeneous product consisting of three forms of thaumatin (Moralejo et al., 1999, 2001; Lombraña et al., 2004). The yield of this production was 5–7 mg/L as seen in Table 4. It can be noticed from these studies that *A. awamori* performed well-compared to the other organisms.

Filamentous fungi have been utilized as an expression platform for screening and production purposes. These fungal-based systems exhibit certain benefits for the high-level secretion of enzymes and the large-scale production of recombinant proteins. However, compared to the bacterial system, filamentous fungi are more complex to understand in terms of their physiology, thereby hindering their potential as efficient factories for heterologous protein production (Su et al., 2012). Moreover, some strains can also be pathogens for humans, animals and plants (Maor and Shirasu, 2005; Cutler et al., 2006; Segal and Walsh, 2006).

### Upstream *Processing*

During a typical *P. pastoris* fermentation to produce heterologous protein, the strain is subjected to a variety of conditions that need to be selected, such as glycerol concentration and feeding for biomass accumulation, methanol feeding for induction of expression, temperature, pH, agitation rate, and dissolved oxygen within the bioreactor. It is vital to understand the influence of the carbon source, promoter and temperature on the metabolism of *P. pastoris*. This information is crucial to avoid the activation of certain metabolic pathways and the production of enzymes that could affect the growth of the organism and recombinant protein production. During *P. pastoris* fermentation, a defined medium containing glycerol as the sole carbon source is preferred for the growth of the strains. After the accumulation of biomass, the production of the protein can be achieved by an induction stage through the addition of methanol.

The whole fermentation process can be explained in three main phases, i.e., glycerol batch phase, glycerol fed-batch phase, and methanol fed-batch phase. Firstly, the strain is grown within a defined medium which consist of glycerol as the sole carbon source. During this period the biomass accumulation takes place, but the heterologous gene is completely repressed. This stage is continued until a high cell density of the desired organism is attained and the carbon source gets depleted. At this point, the heterologous gene expression is repressed. An additional amount of glycerol is still fed into the reactor to facilitate the derepression of the cells and accumulation of biomass (fed-batch phase). This stage will be then followed by the fed-batch phase (transition or induction/production phase) which is initiated by the addition of glycerol-methanol mixture and later by pure methanol (D’Anjou and Daugulis, 2000). During this stage the gene expression takes place and the protein of interest is produced. The conditions inside the reactor can influence the yield of the protein produced and hence needs to be optimized in a way that leads to high cell densities and protein yields. The conditions for optimization in the reactor can be for instance medium composition, pH, temperature, and feeding profiles.

The first cloning of the natural thaumatin II cDNA was performed on the yeast-shuttle vector pPIC9K. This vector has an inducible alcohol oxidase 1 (AOX1) promoter, *S. cerevisiae* prepro α-mating factor secretion signal and a kanamycin resistance gene which can be used for G418 selection (Scorer et al., 1994). Following this, the natural thaumatin II containing vector was transformed into *P. pastoris* GS115 from which approximately 25 mg/l recombinant thaumatin II was obtained. It was observed from further analysis that the obtained recombinant thaumatin contained extra amino acid residues at both ends, i.e., the N- and C- termini. Irrespective of the extra amino acid residues, the resultant recombinant thaumatin still elicited a similar sweetness to the one derived from natural source, demonstrating that the terminal regions do not interfere in the sweetness of the protein (Masuda et al., 2004). *P. pastoris* is seen as an ideal organism for the large-scale production of the recombinant proteins because the medium required for its growth is relatively simple constituting mainly glycerol and methanol as the main carbon sources and other components such as biotin, trace elements and salts. Moreover, they do not involve any components that can contribute to the production of any toxins, hence making it suitable for producing human pharmaceuticals as well. Additionally, the culture medium has less chance to get contaminated by other organisms since *P. pastoris* utilizes a low pH and methanol concentration. Thaumatin cDNA along with the α-factor signal sequence and the Kex2 protease cleavage site were introduced into *P. pastoris* in the study of Ide et al. (2007a). The analysis revealed that the N-terminal consist of two or three unexpected amino acid residues which might be due to the Kex2 protease deficiency resulting in the cleavage of the peptide bond. From the analysis of the N-terminal, it was noticed that some of the unexpected amino acids were attached to it. These amino acids do not affect the threshold value of sweetness of thaumatin (Ide et al., 2007a) but could possibly influence the expression, secretion and structure. The most commonly used *P. pastoris* strains used for the recombinant protein production are mentioned in Alkhalfioui et al. (2011).

The most commonly used *P. pastoris* strain for recombinant protein production is GS115, which is obtained from the wild-type strain NRRL-Y 11430 and has a mutation in the histidinol dehydrogenase gene (HIS4) (Schutter et al., 2009). *S. cerevisiae* and *P. pastoris* hold comparatively similar metabolism with respect to the regulation of the central carbon metabolism and flux ratio profiles for amino acid biosynthesis (Sola et al., 2004). The former has been extensively used for heterologous protein production in the past. Hence, this has the advantage of providing a large amount of data that can be utilized for the optimization of *P. pastoris* culturing in terms of growth and heterologous production. For instance, the minimal media required for the growth of *S. cerevisiae* can be optimized for the biomass production of *P. pastoris*. However, Papanikou and Glick (2009) mention in their study that *P. pastoris* and *S. cerevisiae* do not share the same morphology based on the differences in the Golgi apparatus which could be a reason for *P. pastoris* to secrete the recombinant proteins more efficiently.

Heterologous protein production can be achieved either intracellularly or secreted into the medium by utilizing a secretion signal sequence. The secreted production can be used to avoid problems related to the folding of the proteins and can simplify the purification process compared to the intracellular production. A widely used secretion sequence is the prepro-sequence of the alpha-factor structural gene (MFα1) which is functional in all yeast strains (Brake et al., 1984). The advantage with the extracellular production from *P. pastoris* is that it secretes limited amounts of endogenous proteins and the culture medium does not contain any added proteins. Hence, the largest fraction of the obtained proteins in the medium will be thaumatin (Tschopp et al., 1987b; Bar et al., 1992).

Table 5 showcases the use of different strains of *P. pastoris* for the production of thaumatin utilizing specific plasmids. Masuda et al. (2004) was the first research to report the expression of thaumatin by a *P. pastoris* strain. Natural mature thaumatin II was produced at a yield of about 25 mg/L and was found to elicit a sweet taste, comparable to that of thaumatin from the natural source. Four years later, the thaumatin I variant was produced in *P. pastoris* by Ide et al. (2007a). The authors also attempted to determine common features between proteins eliciting a sweet taste but were unsuccessful. At the same time, Ide et al. (2007b) determined that the pre-sequence has an advantage for the secretion of thaumatin into the culture medium. This sequence is identical to the natural secretion signal. In Ohta et al. (2008), thaumatin mutants were produced to determine the amino acid residues that deliver the sweet taste. Two key amino acid residues (R82 and K67) were identified that played a key role in binding to the taste receptors. A further study on the intensely sweet taste of thaumatin II was made by Masuda et al. (2014). Masuda et al. (2010) used an expression vector that contained three copies of the thaumatin I gene to drive up the yield to 100 mg/L. Healey et al. (2017) tested the co-expression of thaumatin with disulfide isomerase to facilitate protein folding. This led to a doubling in the production rate of thaumatin. This evolution shows the large advances that have been made in understanding the sweetness and microbial production of thaumatin.

**Table 5:** Recombinant thaumatin production utilizing various strains of *Pichia pastoris*.

Thaumatin form	Pichia strain	Gene	Yield	Literature
Thaumatin II	GS115	Natural mature	25 mg/L	Masuda et al., 2004
Thaumatin I	X-33	Natural mature	30 mg/L	Ide et al., 2007a
Thaumatin I	X-33	Natural pre/pro	60 mg/L	Ide et al., 2007b
Thaumatin I	X-33	Natural pre	15 mg/L	Ohta et al., 2008
Thaumatin I	X-33/SMD1168H	Multiple copies	100 mg/L	Masuda et al., 2010
Thaumatin II	X-33	Natural pre	30–50 mg/L	Masuda et al., 2014
Thaumatin I	PPS-9010	Natural mature + chaperone	50 mg/L	Healey et al., 2017

Alcohol oxidase (AOX) is the enzyme that catalyzes the first step in the methanol utilization pathway (Hartner and Glieder, 2006). In this step, methanol is oxidized to formaldehyde while reducing O_2_ and H_2_O. Following this step, the formaldehyde either gets oxidized to CO_2_, thereby giving rise to two molecules of NADH, or gets condensed with xylulose 5-phosphate and then is converted to dihydroxyacetone and glyceraldehyde 3-phosphate within the methanol utilization pathway (Hartner and Glieder, 2006). *P. pastoris* contains two genes AOX1 and AOX2 that encodes two enzymes with AOX activity (Cereghino and Cregg, 2000). The former is controlled by the strong pAOX1 promoter and the latter by the weaker pAOX2 promoter. The AOX1 gene constitutes for most of the alcohol oxidase activity within the cell (Ellis et al., 1985; Tschopp et al., 1987a; Cregg et al., 1989). *P. pastoris* consists of three phenotypes which are involved with the methanol utilization. These include (i) methanol utilization plus (Mut^+^), where both genes are active and intact, (ii) methanol utilization slow (Mut^s^) where AOX1 is knocked out and (iii) methanol utilization minus (Mut^-^), where both AOX genes are knocked out when methanol is used as the sole carbon source (Cereghino and Cregg, 2000). In the Mut^-^ strain methanol acts more like an inducing agent for the production of the recombinant proteins since they are unable to metabolize it Chiruvolu et al. (1997). The Mut^+^ strains showcased a maximum specific growth rate of 0.15 h^-1^ at an excess concentration of methanol as substrate and a temperature of 30°C (Kobayashi et al., 2000).

### Downstream *Processing*

Purification and recovery of recombinant proteins is a crucial step in a bioprocess. This may include a series of techniques depending on the protein of interest and the mode of production. In the case of downstream processing following an intracellular production, the washed and resuspended cell pellets are broken by vortexing with glass beads and the supernatant containing the cytosolic proteins is recovered by centrifugation. The concentration of the proteins could be determined through the bicinchoninic acid assay (Ide et al., 2007a). One of the major limitations associated with the recovery of heterologous proteins from the *P. pastoris* system would be proteolysis of the secreted proteins and cell death within the bioreactor. This can result in lower quality of the product after the downstream processing (Jahic et al., 2006).

A simplified purification process can be applied when using secreted production. This secreted production is often possible for foreign proteins that are secreted naturally by their native hosts. This comes with the requirement for a signal sequence to guide the protein through the secretory pathway. The native secretion signal sequences have been used extensively. However, the most successful has been identified as the *S. cerevisiae*α-factor prepro-peptide. This signal sequence includes a 19-amino acid signal (pre) sequence and a 66-residue (pro) sequence consisting of *N*-linked glycosylation sites and a Kex2 endopeptidase processing site (Kurjan and Herskowitz, 1982). Brake et al. (1984) elaborates on the three major steps involved in processing the signal sequence. If the protein secretion is successful, the next important step is to recover the protein and attain it in purest form. The recovery of the protein of interest in the most economical way requires an advanced downstream processing and purification. Proteins attained through secreted production facilitate an easier recovery through filtration-based purification and concentration.

Several papers discuss on the possible analytical methods that can accommodate the characterization of thaumatin. For instance, Masuda (2016) elaborates in his paper on the use of an SP-Sephadex C-25 column to separate the dialyzed culture supernatant. Followed by this step, the desired fraction was precipitated using ammonium sulfate. It is further dialyzed and applied to a Toyopearl HW-50F column and eluted with a buffer. The attained protein is checked for its purity by utilizing the SDS-PAGE and native PAGE techniques and quantified using the bicinchoninic acid method. The purification of the protein can depend on the type of protein and tag used.

One of the common methods adopted for the purification of recombinant proteins is through chromatography, specifically affinity chromatography because of their high specificity. Tags are used for improving the solubility and for facilitating affinity purification. These tags are small structures with short sequences of just 3–4 amino acids that can be added to the recombinant protein and hence allows to capture the proteins with ease. Apart from these, some fusion tags can be useful for a range of applications such as labeling for imaging studies, localization studies, detection, quantification, protein–protein interaction studies, subcellular localization or transduction and many others (Malhotra, 2009). Hence, the selection of an appropriate tag while designing the expression constructs is an important step and often puts the researcher in a dilemma to choose. The common tags associated with the recombinant protein production are histidine tag, glutathione-*S*-transferase, maltose binding protein, etc. The tags can be positioned at the N- or C- terminus of the protein of interest. However, placing them at the N-terminal comes with the advantage of solubilizing the target proteins (Sachdev and Chirgwin, 1998) and the easy removal of the tag. Since these tags have an influence on the structure and function of the end protein, it is necessary to remove them during the purification step with the usage of endoproteases such as tobacco etch virus protease or thrombin.

## Perspectives

Several biopharmaceuticals are nowadays produced by the utilization of technologically advanced microbial and mammalian cell biosystems. This technique provides advantages for the production of important recombinant pharmaceuticals in a safe and abundant quantity. The utilization of a high number of engineered strains of *P. pastoris* and the designing of metabolic pathways for the enhanced production of the heterologous proteins have been seen in recent years. Today, *P. pastoris* is extensively utilized as a successful expression system for both industrial and research purposes (Cereghino and Cregg, 2000; Macauley-Patrick et al., 2005). The use of recombinant DNA helps in the amplification and cloning of important genes within *P. pastoris*. As such, there is a plethora of opportunities for the production of protein of interest with this emerging technique. The metabolism includes several coupled and interconnecting reactions. The term metabolism refers to a reaction comprising the conversion of a molecule into another molecule or molecules within a defined pathway. A cell includes numerous pathways of such reactions which are independent and that can be coordinated with the presence of enzymes. Understanding the metabolic reactions gives insights on the information related to the fluxes. This information leads a path for the establishment of the metabolic model which can serve as a tool for setting up an optimized bioreactor process. Such a tool can be advantageous to predict the parameters influencing the process or genetic modifications on strain behavior (Kerkhoven et al., 2014; Cankorur-Cetinkaya et al., 2017).

Considering the increased interest in *P. pastoris*, methods for increasing the production of recombinant proteins with this host are of high relevance (Zhang et al., 2005). This can be achieved by genetically engineering the metabolism of the microorganism in such a way that the product yield is maximized. In general, the processes involved in a microbial metabolism are complex and a significant effort is required to study these processes. Specifically, for *P. pastoris*, there are still some limitations persisting for the engineering of this non-conventional yeast, due to a lack of genome editing tools and incomplete knowledge on their cell behavior when compared with the yeast platforms such as *S. cerevisiae*. As such, this line of work comes with the limitations of time consumption, high cost and production of noisy data. Therefore, another method to be adopted can be the development of mathematical models which have the ability to characterize the various phenotypes their culturing behavior (Sidoli et al., 2004; Royle et al., 2013).

Even though genetic engineering is a valuable first step, on a large-scale production of these compounds, a further optimization of the process based on carbon and nitrogen sources, feeding profiles, aeration levels and so on will be necessary as well. In order to perform this optimization in a systematic manner, it is necessary to learn how the behavior of *P. pastoris* during culturing can be predicted. Therefore, the key factors influencing microbial growth and protein production should be determined and the relationships between them should be captured in a mathematical model. An appropriate model structure should describe effects that limit recombinant protein production such as cell viability, metabolite accumulation, metabolite toxicity, substrate requirement, and substrate inhibition.

### Systems Biology

Systems biology is an approach which helps in studying the interactions within biological systems. It facilitates in understanding the behavior of systems involving several enzymes, metabolites and metabolic pathways or signaling networks. This understanding is obtained with the aid of mathematical models and computational techniques (Vidal, 2009). Hence, it can be deduced from available literature that systems biology adopts a holistic approach involving a complete system in comparison to molecular biology that involves subsystems through *in vitro* studies (Kell and Oliver, 2003). This approach relies on the vast amount of biological information that is available on several biological systems. However, the integration of significant amounts of high-quality data is often still needed to obtain the desired insights.

The utilization of microbial cell factories within industrial microbiology involves a constant process of strain engineering and optimization of the bioprocessing conditions. The main target with respect to the production of thaumatin would be to accommodate the maximum microbial production rate. For this, an optimal environment needs to be provided toward the culturing of the microorganism on one hand and the production of thaumatin on the other. With this respect, the media composition has a major influence on the overall performance of the bioprocess. Systems biology provides highly effective tools such as flux balance analysis and metabolic flux analysis for the optimization of media composition. However, both require that information on the metabolic network of the microorganism and the latter also requires a significant amount of experimental data. There has been sufficient data available already from studies depicting that an optimized nutritional medium is required for the organism to enhance the recombinant protein production. However, in order to obtain a deeper knowledge on the actual effect of media composition on cell growth and product formation, additional data is still required. The data itself can be from cell physiology (Rehberg et al., 2013), extracellular metabolites (Genzel et al., 2004), intracellular metabolites (Ritter et al., 2006), and enzyme activities (Janke et al., 2010). Most importantly, this data should provide information on the metabolic response of the selected organism. This response can be quantified using mathematical modeling techniques.

The inclusion of microscopic models has the advantage of resulting in more mechanistic and generic predictive models when compared to the macroscopic models that do not take into account the underlying mechanisms that cause a certain response. For example, macroscopic models will simply describe the production rate of the end-product based on some key influencing factors whereas microscopic models will consider in more detail the metabolic reactions that lead to the production of this product. Despite the fact that the metabolism of, e.g., *P. pastoris*, has been studied over the years, many parts of it still remain unclear. For instance, the secondary metabolism is very diverse in addition to the complexity existing between the connections in the various reactions thereby resulting in an extremely dense biological network. The *in silico* reconstructions of the metabolism can be achieved from the genome-scale metabolic models. Such a model helps in the linkage between the genotype and metabolic capability.

### Genome-*Scale Metabolic Models*

When looking into the traditional techniques used for modeling bioreactor processes, they are derived from mass balances of biomass and growth limiting carbon source. Similar model types can be utilized for the bioproduction of *P. pastoris*. These classical approaches are based on a system with ordinary differential equations or their explicit form. However, this information alone does not provide mechanistic knowledge on the cell. Further advancements have taken place with respect to model development through the integration of information on the metabolic level. These types of studies have been performed as well for *P. pastoris* strains. As such, the emergence of the implementation of genome-scale metabolic models has magnified the knowledge on the cell behavior. The first such model constructed for a eukaryotic organism was for *S. cerevisiae*. Now, there have been many studies involved in the reconstruction and expansion of the metabolic networks for yeasts. The various reconstructions of the genome-scale metabolic network models for *P. pastoris* are given in Table 6.

**Table 6:** Available genome-scale metabolic models for *P. pastoris*.

Model	Reactions	Metabolites	Reference
iPP668	1354	1177	Chung et al., 2010
PpaMBEL1254	1254	1058	Sohn et al., 2010
iLC915	1423	1302	Irani et al., 2015
iMT1026	2035	1689	Tomàs-Gamisans et al., 2016
iRY1243	2407	1740	Ye et al., 2017
iFS670	1383	1195	Saitua et al., 2017

The use of metabolic models helps in improving the production of target compounds and for uncovering new information on the biological systems. Hence, these models play a significant role in the design and optimization of bioprocesses. This way, a more quantitative and systematic framework that has the potential to improve the value and reliability can be achieved. It is noticed from recent literature that the engineering approach toward cell metabolism boosts the productivity of recombinant proteins. This is evident from the high number of pathway designs and engineered constructs that are available for *P. pastoris* today. A full metabolism involves numerous reactions and hence, utilizing mathematical models contributes toward a better understanding of this complex process.

The optimization of fermentation processes requires the selection of numerous conditions such as pH, temperature, oxygen concentration and nutrient supply (Cos et al., 2006; Jahic et al., 2006; Calik et al., 2015). The use of a mathematical model is significant for the conversion of the available experimental data into new insight (Nielsen, 2017) and helps in getting information in a concise manner. However, several aspects on the true mechanics of the bioprocess still remain unknown, limiting the possibility to build a model that depicts the full set of cellular processes for a given microorganism. Most of the studies related to the metabolic modeling of *P. pastoris* are based rather on the influence of protein production on the metabolism than the prediction on specific efficiency. Hence, the genome-scale metabolic models seem to be a very valuable tool for the optimization of biochemical production processes that are still not used to their full potential.

## Conclusion

Sweet proteins are seen to a have great potential today in various applications and are expected to rise to a level capable enough to replace sucrose in several applications. Due to their low-caloric feature and great qualities as a sugar replacer, the consumers have perceived these natural sweeteners with a positive outlook. Thaumatin specifically is emerging as a strong alternative to sucrose and other synthetic replacers in the market today. Although thaumatin has been studied by various researchers over the past 30 years, there is still much work to be done in order to improve its production via the biochemical routes. It is evident from the literature that biological products are emerging as a promising applicant within the food industry. Hence, there is great potential for future research that focuses on the use of advanced computational techniques for the optimization of thaumatin bioproduction.

## Author Contributions

JJ delivered the main contribution in reviewing literature and writing, in close collaboration with SA and PN. JI supervised the writing of this review. The general content and lay-out of this publication were determined by all authors.

## Conflict of Interest Statement

The authors declare that the research was conducted in the absence of any commercial or financial relationships that could be construed as a potential conflict of interest.

## Footnotes

1 apps.who.int/iris/bitstream/10665/149782/1/9789241549028_eng.pdf

2 http://www.fda.gov/food/ingredientspackaginglabeling/foodadditivesingredients/ucm397725.htm

3 http://www.fda.gov/food/ingredientspackaginglabeling/foodadditivesingredients/ucm397725.htm

4 www.globaldata.com/global-data-2015

5 https://www.coherentmarketinsights.com/market-insight/recombinant-protein-market-1516
